# Supplementary figures and images for: Improved smart city security using a deep maxout network-based intrusion detection system with walrus optimization
Source: PeerJ Comput Sci. 2025 Mar 31;11:e2743. doi: 10.7717/peerj-cs.2743 (PMC12190705; doi:10.7717/peerj-cs.2743)

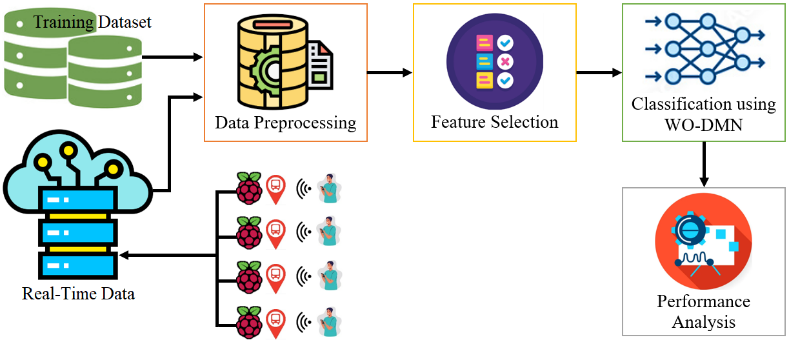

Supplement: Supplemental Information 1 [file peerj-cs-11-2743-s001.zip › DMN-main/1.png]

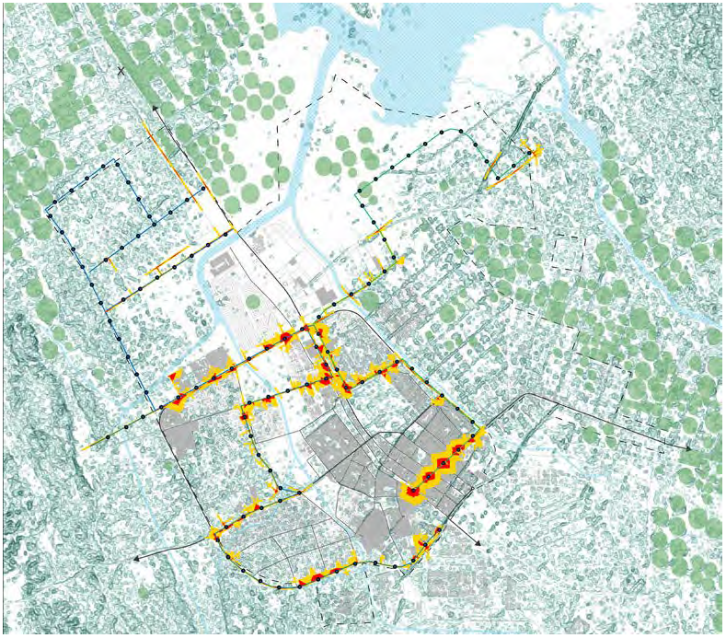

Supplement: Supplemental Information 1 [file peerj-cs-11-2743-s001.zip › DMN-main/2.png]

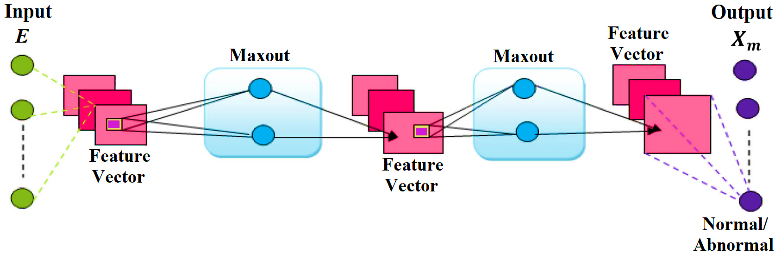

Supplement: Supplemental Information 1 [file peerj-cs-11-2743-s001.zip › DMN-main/3.png]
